# Aortic calcification burden predicts deterioration of renal function after radical nephrectomy

**DOI:** 10.1186/s12894-017-0202-x

**Published:** 2017-02-06

**Authors:** Ken Fukushi, Shingo Hatakeyama, Hayato Yamamoto, Yuki Tobisawa, Tohru Yoneyama, Osamu Soma, Teppei Matsumoto, Itsuto Hamano, Takuma Narita, Atsushi Imai, Takahiro Yoneyama, Yasuhiro Hashimoto, Takuya Koie, Yuriko Terayama, Tomihisa Funyu, Chikara Ohyama

**Affiliations:** 1 Department of Urology, Hirosaki University Graduate School of Medicine, 5 Zaifu-chou, Hirosaki, 036-8562 Japan; 2 Department of Advanced Transplant and Regenerative Medicine, Hirosaki University Graduate School of Medicine, Hirosaki, Japan; 3 Department of Urology, Oyokyo Kidney Research Institute, Hirosaki, Japan

**Keywords:** Aortic calcification, Chronic kidney disease, Radical nephrectomy, Renal cell carcinoma, Renal function

## Abstract

**Background:**

Radical nephrectomy for renal cell carcinoma (RCC) is a risk factor for the development of chronic kidney disease (CKD), and the possibility of postoperative deterioration of renal function must be considered before surgery. We investigated the contribution of the aortic calcification index (ACI) to the prediction of deterioration of renal function in patients undergoing radical nephrectomy.

**Methods:**

Between January 1995 and December 2012, we performed 511 consecutive radical nephrectomies for patients with RCC. We retrospectively studied data from 109 patients who had regular postoperative follow-up of renal function for at least five years. The patients were divided into non-CKD and pre-CKD based on a preoperative estimated glomerular filtration rate (eGFR) of ≥60 mL/min/1.73 m^2^ or <60 mL/min/1.73 m^2^, respectively. The ACI was quantitatively measured by abdominal computed tomography before surgery. The patients in each group were stratified between low and high ACIs. Variables such as age, sex, comorbidities, and pre- and postoperative renal function were compared between patients with a low or high ACI in each group. Renal function deterioration-free interval rates were evaluated by Kaplan-Meier analysis. Factors independently associated with deterioration of renal function were determined using multivariate analysis.

**Results:**

The median age, preoperative eGFR, and ACI in this cohort were 65 years, 68 mL/min/1.73 m^2^, and 8.3%, respectively. Higher ACI (≥8.3%) was significantly associated with eGFR decline in both non-CKD and pre-CKD groups. Renal function deterioration-free interval rates were significantly lower in the ACI-high than ACI-low strata in both of the non-CKD and pre-CKD groups. Multivariate analysis showed that higher ACI was an independent risk factor for deterioration of renal function at 5 years after radical nephrectomy.

**Conclusions:**

Aortic calcification burden is a potential predictor of deterioration of renal function after radical nephrectomy.

**Trial registration:**

This study was registered as a clinical trial: UMIN000023577

**Electronic supplementary material:**

The online version of this article (doi:10.1186/s12894-017-0202-x) contains supplementary material, which is available to authorized users.

## Background

Various studies have shown that radical nephrectomy for renal cell carcinoma (RCC) is an independent risk factor for the development of chronic kidney disease (CKD) [1, 2]. A radical nephrectomy remains the standard treatment for patients, depending on factors such as tumor size, tumor location, and comorbidities, nevertheless CKD increases the risk of cardiovascular events and overall mortality [3]. Although it is suggested that CKD as a consequence of surgery (such as nephrectomy) may be associated with a relatively lower risk of progression and mortality than CKD attributed to medical disorders (such as hypertension, type 2 diabetes, and/or cardiovascular disease) [4, 5], any CKD is associated with an increased risk of cardiovascular events and all-cause mortality in large, population based studies [6–8]. Therefore, when discussing the therapeutic option of radical nephrectomy, it is essential to estimate the risk of postoperative deterioration of renal function. Potential factors associated with severe postoperative renal impairment have been reported, such as old age, preexisting stage 3 CKD, hypertension, type 2 diabetes, and cardiovascular disease [1–3].

Aortic calcification is one of the sequelae of aortic degeneration and has recently been considered a major complication and independent risk factor for coronary artery disease, heart failure, and stroke [9, 10]. Several studies have addressed the clinical importance of arterial calcification in patients at high risk for cardiovascular disease; however, only a few studies have demonstrated the impact of arterial calcification on renal function [11–13]. In addition, the clinical relevance of the aortic calcification index (ACI) with regard to postoperative renal function outcome in patients undergoing radical nephrectomy has not been well studied. Therefore, we hypothesized that preexisting aortic calcification may play a crucial role in the deterioration of renal function after radical nephrectomy and investigated the contribution of the ACI in predicting postoperative renal function in such patients. This study was registered as a clinical trial: UMIN000023577 (http://rctportal.niph.go.jp/en/detail?trial_id=UMIN000023577).

## Methods

### Patient selection and variables evaluated

Between January 1995 and December 2012, we performed 511 consecutive radical nephrectomies in patients with RCC. Of those, we excluded 95 patients who deceased within five years, 165 patients without baseline abdominal computed tomography (CT) prior to radical nephrectomy, and 142 patients without postoperative regular assessment of renal function for five years after the surgery. The remaining 109 patients were included in the present study. Blood tests including renal function were routinely performed at least once a year after radical nephrectomy. The estimated glomerular filtration rate (eGFR) was calculated using the Modification of Diet in Renal Disease equation for Japanese patients [14].

The patients were divided in two groups depending on the preoperative eGFR, a non-CKD group with an eGFR of ≥60 mL/min/1.73 m^2^　and a pre-CKD group with an eGFR <60 mL/min/1.73 m^2^.

**Fig. 1:**
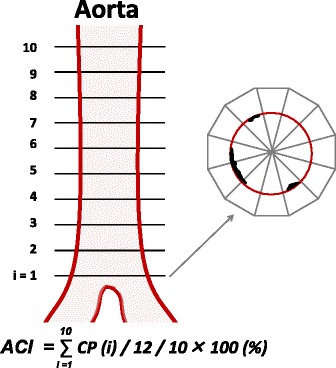
Patient selection and measurement of aortic calcification index (ACI). Aortic calcification was quantitatively measured using preoperative abdominal computed tomography images, evaluating 10 slices scanned at 5-mm intervals above the abdominal aortic bifurcation. Each slice was divided in 12 sectors and the numbers of sectors with calcification were counted. The ACI was calculated by averaging the percentage of calcium-positive sectors in each slice

Using abdominal CT images (TSX-301B, Toshiba Medical Systems Corp., Ohtawara, Japan, or CT750HD, GE Healthcare Japan, Tokyo, Japan), the ACI was quantitatively measured by scanning 10 slices of the aorta at 5-mm intervals above the bifurcation of the common iliac arteries as previously described [15]. Intimal calcification was scored in each of 12 radial sectors of each slice and reported as a percentage. For example, if 5 of 12 sectors were calcified in section 1, it was scored as 5/12 = 41.7% (Fig. 1). The ACI (%) was calculated as the average intimal calcification values of sections 1–10. ACI measurement was performed by an investigator who was blind to patients’ clinical parameters.

The patients in each group were stratified depending on whether their ACIs were higher or lower than 8.3%, based on the median value of ACI in this cohort. Variables including age; sex; history of hypertension, type 2 diabetes, or cardiovascular disease; clinicopathologic data on the renal cancer; and renal function before and for 5 years after surgery were compared between patients with low and high ACI in each group. Hypertension was defined as taking any antihypertensive medications or preoperative systolic and diastolic blood pressure measurements of >140 and >90 mmHg. Diabetes was defined a history of type 2 diabetes or meeting the relevant diagnostic criteria and requiring glycemic control. Cardiovascular disease was defined as a positive history of cardiac surgery, angina, myocardial infarction, or stroke or taking any cardiotonic agents or coronary vasodilators.

The interval until the progression of CKD was measured from 3 months after surgery until the eGFR decreased in the non-CKD group to <60 ml/min/1.73 m^2^ (defined as CKD3-free interval) or in the pre-CKD group to <45 ml/min/1.73 m^2^ (defined as CKD3B-free interval).

### Statistical analysis

Statistical analysis was performed using SPSS v. 22.0 (IBM Corporation, Armonk, NY, USA) and GraphPad Prism v. 5.03 (GraphPad Software, San Diego, CA, USA). Categorical variables were reported as percentages and compared using Fisher’s exact test. Quantitative data were expressed as medians with quartiles 1 and 3 (Q1, Q3). Differences between the groups were compared using a *t* test for normally distributed data or Mann—Whitney *U* test for data with a non-normal distribution. Probability (*P*) values of <0.05 were considered statistically significant. Renal function deterioration-free intervals were evaluated by Kaplan-Meier analysis. Independent factors associated with the renal function deterioration-free interval were identified by multivariate analysis using a Cox regression model. Hazard ratios (HR) with 95% confidence intervals (95% CI) were calculated after adjusting for potential confounders. Multivariate logistic regression analysis was performed to identify significant risk factors for eGFR loss >30% after radical nephrectomy at 5 years. Odds ratios (OR) with 95% CI were calculated after adjusting for potential confounders. Liner regression analysis was performed to develop a 5-year eGFR prediction formula, and correlation was analyzed using Spearman’s correlation coefficient.

## Results

**Fig. 2:**
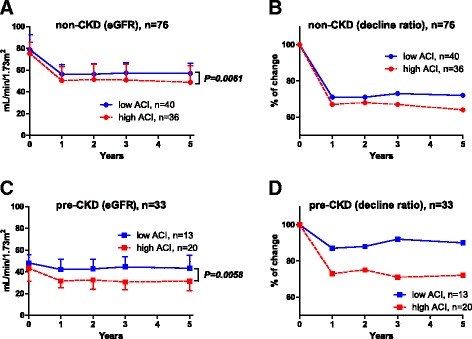
Renal function compared in patients with low and high ACI in non-CKD and pre-CKD groups. Longitudinal evaluation of eGFR reveals significantly poorer renal function in patients with a high than a low ACI in the non-CKD group (**a**) and in the pre-CKD group (**c**). The decline ratios are higher in patients with high rather than low ACI in the non-CKD group (**b**) and in the pre-CKD group (**d**)

Table 1 summarizes patients’ characteristics. The median age, preoperative eGFR, and ACI in this cohort were 65 years, 68 mL/min/1.73 m^2^, and 8.3%, respectively. The median ACI value of 8.3% was used as a cut-off between low and high ACI. Although there were statistically significant differences in ages between the low and high ACI strata in the non-CKD and pre-CKD groups, sex, renal function, comorbidities, and tumor stage did not differ significantly (Table 1). In the non-CKD group, longitudinal evaluation of eGFR revealed that the postoperative median eGFR at 5 years in patients with low and high ACI were 58 and 50 ml/min/1.73 m^2^, respectively (*P =* 0.0061). It also showed significantly poorer renal function in patients with a high than a low ACI in the non-CKD group (Fig. 2a). The decline ratio at 5 years after radical nephrectomy was higher in patients with a high (36%) than a low ACI (28%) (Fig. 2b). Similarly, the postoperative median eGFR at five years in the pre-CKD group was significantly poorer in those with a high rather than a low ACI (32 ml/min/1.73 m^2^ vs. 42 ml/min/1.73 m^2^, respectively, *P =* 0.0058) (Fig. 2c). In the pre-CKD group, the eGFR showed 28% reduction at 1 year after radical nephrectomy in patients with a high ACI, whereas it was 13% in low ACI (Fig. 2d).

**Table 1:** Comparison of clinical and pathological patient’s characteristics between low (<8.3%) and high (≥8.3%) ACI

	non-CKD			pre-CKD		
	low ACI	high ACI	*P value*	low ACI	high ACI	*P value*
n	40	36		13	20	
Age, years	57 (44–63)	67 (62–75)	<0.001	62 (53–71)	76 (74–79)	<0.001
Sex (Male)	26 (65%)	26 (72%)	0.499	9 (69%)	13 (65%)	1.000
Comorbidities						
Hypertension	9 (23%)	11 (31%)	0.426	1 (8%)	9 (45%)	0.050
Diabetes	3 (8%)	7 (19%)	0.177	1 (8%)	3 (15%)	1.000
Cardiovascular disease	2 (5%)	2 (6%)	1.000	0 (0%)	1 (5%)	1.000
Preoperative eGFR	77 (68–87)	74 (67–84)	0.202	50 (42–54)	42 (33–55)	0.152
ACI (%)	0.8 (0.0–5.8)	18 (14–27)	<0.001	0.8 (0.0–3.3)	18 (16–44)	<0.001
Tumor stage						
T1/2/3/4	25/6/7/2	23/3/10/0	0.394	7/4/2/0	13/3/3/1	0.810
Pathological subtype			0.277			1.000
Clear cell	35 (88%)	35 (97%)		12 (92%)	18 (90%)	
Papillary	1 (2.5%)	1 (3%)		1 (8%)	1 (5%)	
Chromophobe	1 (2.5%)	0 (0%)		0 (0%)	1 (5%)	
Others	3 (7.5%)	0 (0%)		0 (0%)	0 (0%)	
Distant metastasis	6 (15%)	3 (8%)	0.494	2 (15%)	3 (15%)	1.000
Tumor recurrence	9 (23%)	5 (14%)	0.334	2 (15%)	2 (10%)	1.000

Median and interquartile range (Q1, Q3) was used for consecutive variables. *ACI* aortic calcification index, *eGFR* estimated glomerular filtration rate

**Fig. 3:**
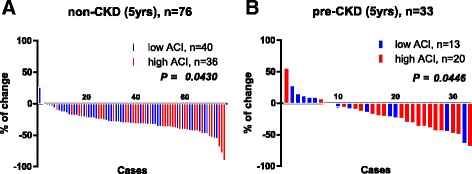
Rate of decline of eGFR over 5 years in non-CKD and pre-CKD groups. Waterfall plots show significant differences between patients with a high or low ACI in the non-CKD group (**a**) and pre-CKD group (**b**). In the pre-CKD group, patients with a high ACI had greater rates of decline (27%) than those with a low ACI (6%) at 5 years after radical nephrectomy

In terms of the rates of decline in eGFR, significant differences were observed in both groups between patients with low and high ACI (Fig. 3a, b). The median rate of decline at 5 years was significantly higher in patients with a high (32%) versus low ACI (28%) in the non-CKD group (*P* = 0.0430) (Fig. 3a). Similarly, patients with high ACI in the pre-CKD group exhibited remarkably higher rates of decline (27%) than did those with low ACI (6%) at 5 years after radical nephrectomy (*P* = 0.0446) (Fig. 3b).

**Fig. 4:**
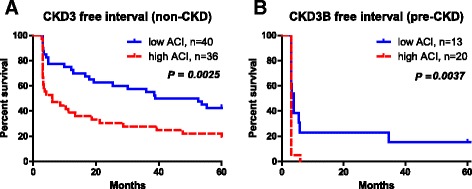
CKD stage progression-free interval after radical nephrectomy. The CKD stage progression-free interval rates are significantly lower in the patients with high ACI after radical nephrectomy in the non-CKD group (**a**) and in the pre-CKD group (**b**). CKD stage progression is defined as CKD stage 0–2 progressing to stage 3 in the non-CKD group and CKD stage 3A progressing to 3B in the pre-CKD group

In the non-CKD group, CKD stage 3-free interval was significantly shorter in patients with high ACI than in those with low ACI (*P* = 0.0025) (Fig. 4a). Five-year CKD stage 3-free rates were 19.4% and 42.5% in patients with high and low ACI, respectively. Similarly, in the pre-CKD group, the CKD stage 3B-free intervals were significantly shorter in patients with high than with low ACI (*P* = 0.0037) (Fig. 4b) Five-year CKD stage 3-free rates were 0% and 15.4% in patients with a high or low ACI, respectively.

**Fig. 5:**
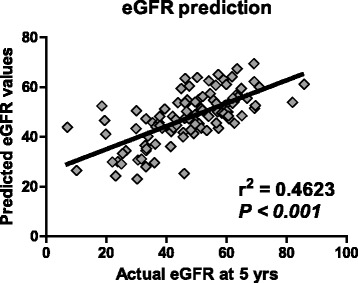
The 5-year eGFR prediction model after radical nephrectomy. The formula to predict 5-year eGFR was developed by linear regression analysis including three key factors (age, preoperative eGFR, and ACI). The formula is: Y (estimated 5 years eGFR) = (0.351 x preoperative eGFR) + (−0.232 x age) + (−0.208 x ACI) + 41.758. The predicted 5-year eGFR is significantly correlated with actual eGFR at 5 years after radical nephrectomy

Multivariate Cox regression analysis revealed that age, preoperative eGFR, and ACI were independent risk factors for CKD progression (Table 2). Multivariate logistic regression analysis revealed that age, preoperative eGFR, and ACI were independent risk factors for eGFR loss >30% at five years after radical nephrectomy (Table 3). Based on the significant variables, we developed a formula by linear regression analysis to predict eGFR at five years after radical nephrectomy: Y (estimated 5-year eGFR) = (0.351 x preoperative eGFR) + (−0.232 x age) + (−0.208 x ACI) + 41.758. The correlation between actual eGFR and predicted values were significant (*P <* 0.001, r^2^ = 0.4623) (Fig. 5). The minimal data set of the present study is available in Additional file 1: Dataset S1.

**Table 2:** Multivariate Cox regression analysis for risk factors for stage 3 CKD (eGFR < 60 mL/min/1.73 m^2^) in non-CKD group, or stage 3B CKD (eGFR < 45 mL/min/1.73 m^2^) in pre-CKD group

Cox regression analyses	Risk factor	*P value*	HR	95% CI
Age, years	Continuous	0.224	1.01	0.99–1.04
Sex	Male	0.520	0.85	0.53–1.38
Diabetes	Positive	0.936	0.97	0.51–1.86
Cardiovascular disease	Positive	0.542	1.35	0.52–3.51
ACI (%)	Continuous	0.044	1.02	1.00–1.03
Metastatic disease	Positive	0.053	0.47	0.22–1.01
Preoperative eGFR (ml/min/1.73 m^2^)	Continuous	<0.001	0.97	0.96–0.99

*ACI* aortic calcification index, *CKD* Chronic kidney disease, *eGFR* estimated glomerular filtration rate

**Table 3:** Multivariate logistic regression analyses for risk factors for loss of renal function greater than 30% at 5 years after nephrectomy

Logistic regression analysis	Risk factor	*P value*	OR	95% CI
Age, years	Continuous	0.137	1.03	0.99–1.08
Sex	Male	0.714	1.20	0.46–3.14
Diabetes	Positive	0.202	0.40	0.10–1.63
Cardiovascular disease	Positive	0.735	1.36	0.23–8.19
ACI (%)	Continuous	0.016	1.05	1.01–1.09
Metastatic disease	Positive	0.763	0.81	0.21–3.18
Preoperative eGFR (ml/min/1.73 m^2^)	Continuous	<0.001	1.07	1.03–1.10

*ACI* aortic calcification index, *CKD* Chronic kidney disease, *eGFR* estimated glomerular filtration rate

## Discussion

In the present study, we demonstrated that a higher burden of aortic calcification was an independent risk factor for deterioration of renal function in patients after radical nephrectomy. In addition, the postoperative decline in renal function was significantly worse in patients with a high ACI regardless of their preoperative eGFR. Multivariate analysis revealed that the association of ACI with deterioration of renal function was statistically significant. Our findings suggest that a patient’s ACI may be useful in predicting renal function after unilateral nephrectomy.

Because aortic calcification is one of the results of aortic degeneration, we hypothesized that it might predict deterioration of renal function after unilateral nephrectomy. Indeed, our previous study suggested that the burden of aortic calcification has impact on poor postoperative renal function in renal transplant patients [12], as well as with persistent hypertension after unilateral adrenalectomy in patients with aldosterone-producing adenomas [16]. Although the mechanisms by which aortic calcification might influence glomerular microcapillary degeneration remain unclear, it is not hard to anticipate that vascular damage occurs first and more severely in small vessels such as afferent arterioles and glomeruli.

Risk factors for aortic calcification include older age, higher systolic blood pressure, smoking, increased oxidative stress, dyslipidemia, the presence of diabetes and/or cardiovascular disease, and CKD [17, 18]. However, as all these factors are correlated with each other, the association is confusing, similar to asking whether the chicken or the egg comes first. The element that is common to all such risk factors is chronic, low grade, systemic inflammation, which has been defined as a metabolic syndrome [19]. A recent large study revealed that aortic valve calcification involves inflammatory, lipid, and mineral metabolism pathways [20]. In addition, inflammation and oxidative stress have been linked to vascular calcification in patients with CKD [18]. Therefore, chronic, low grade, systemic inflammation may accelerate arterial degeneration, resulting in arterial calcification and reduced renal reserve. Further studies are necessary to address the detailed association between aortic calcification, inflammation, oxidative stress, and CKD.

Aortic calcification was once believed to be a simple passive process of aging. However, it is now recognized as a complex and highly regulated systemic process that involves the activation of cellular signaling pathways, genetic factors, and hormones. It had been assumed to be irreversible, and no agents were available to prevent it. However, a recent study reported that policosanol has the potential to inhibit vascular calcification [21]. Elseweidy et al. reported that treating diabetic hyperlipidemic rats with policosanol, omega-3 fatty acids, and atorvastatin for eight weeks significantly increased high-density lipoprotein cholesterol (HDL-C) and vitamin D, decreased the number of aortic vacuoles, and inhibited the calcification process. Of the agents used, policosanol induced more remarkable reduction in the density and number of foam cells and improved intimal lesions of the aorta more than atorvastatin. The key effects of policosanol are inhibition of inflammation, oxidative stress, and calcium deposition, all of which are essential pathways in aortic calcification.

In the present study, we found that patients in the pre-CKD group who had a higher ACI had a marked decrease in renal function (Fig. 2d). Because older patients were included in this group, the result might reflect selection bias. However, multivariate analysis showed that preoperative eGFR and ACI were independent predictors’ deterioration of renal function after controlling for other patient variables. Therefore, our findings suggest that patients with RCC who have both preoperative CKD and a high ACI are at significant risk for severe deterioration in renal function after unilateral nephrectomy. Although our observation needs further investigation, aortic calcification appears to be a potential surrogate marker for diminished renal reserve after unilateral nephrectomy.

Several limitations need to be acknowledged. First, selection of RCC patients with renal function for 5 years is a main limitation of our study. The small sample size and single-institution retrospective design prevent definitive conclusions on the influence of aortic calcification on postoperative renal function. The fact that only one observer calculated the ACI parameter could be another limitation. Significant differences in ages between the low and high ACI stratified groups in the non-CKD and pre-CKD groups are a strong limitation that restricts the value of the findings. We could not exclude the multiple collinearity among variables including age, eGFR, Hypertension, and ACI. Definition of hypertension is not suitable for detection postoperative renal impairment. Whether our findings are generalizable to non-Asian populations is also unclear. In addition, we were unable to include in the analysis certain other established risk factors for renal dysfunction, such as cigarette smoking, dyslipidemia, proteinuria, blood pressure control, and medications.

Second, our study did not assess the influence of aortic calcification on cardiovascular events or overall survival after radical nephrectomy in patients with RCC. Because CKD resulting from surgery may not increase mortality as much as CKD that is secondary to medical conditions, [5] the association between a decline in renal function after radical nephrectomy and mortality is under debate. Recent research suggests that survival is better with CKD occurring after surgery rather than with other diseases, particularly if the postoperative eGFR is greater than 45 ml/min/1.73 m^2^, whereas patients with preexisting CKD are at risk of a significant decline in renal function after surgery [8]. Therefore, further large-scale, long-term studies are required to resolve these issues.

Despite these limitations, the strength of this study is its novel finding of the association between aortic calcification and deterioration renal function after radical nephrectomy. Using a non-invasive modality of measuring ACI, we were able to demonstrate an independent association between aortic calcification and postoperative renal impairment. Our findings may assist in recognizing patients who are at high risk of CKD after radical nephrectomy, which will help to inform the preoperative discussion of therapeutic options.

## Conclusions

In conclusion, the aortic calcification burden is a potential predictor for postoperative renal impairment. It may be useful to identify patients who are at high risk for renal impairment after radical nephrectomy.

## Additional file

Additional file 1: Dataset-S1, Simple data set. Simple data set is available in supplemental file (Dataset S1, MS Excel). Mean (± standard deviation) and median (interquartile range) age were 63 ± 13 and 65 (55–73) years old, respectively. Median height (cm) and body weight (kg) were 161 (155–167) and 63 (54–72), respectively. (XLSX 32 kb)

## Abbreviations

ACI: Aortic calcification
CKD: Chronic kidney disease
CT: Computed tomography
eGFR: Estimated glomerular filtration rate
HDL-C: High-density lipoprotein cholesterol
HR: Hazard ratio
OR: Odds ratio
Q1: First quartile
Q3: Third quartile
RCC: Renal cell carcinoma
